# Supplementary material for: Efficacy of the Piperidine Nitroxide 4-MethoxyTEMPO in Ameliorating Serum Amyloid A-Mediated Vascular Inflammation
Source: Int J Mol Sci. 2021 Apr 27;22(9):4549. doi: 10.3390/ijms22094549 (PMC8123591; doi:10.3390/ijms22094549)
Supplement: Supplementary file 1 [file ijms-22-04549-s001.zip › ijms-1194052-supplementary.pdf]

Table S1. Primer sequences

Primer	Forward Sequence (5′-3′)	Reverse Sequence (3′- 5′)	NCBI Accession number
**GAPDH**	CTGGGCTACACTGAGCACC	AAGTGGTCGTTGAGGGCAATG	NM_001357943.2
**VCAM-1**	AATGCCTGGGAAGATGGTCG	GATGTGGTCCCCTCATTCGT	NM_001078.4
**ICAM-1**	TATAAAGGATCACGCGCCCC	AACAACTTGGGCTGGTCACA	NM_000201
**E-Selectin**	CTGGCAGTTCCGGGAAAGAT	AGCCAGAGGAGAAATGGTGC	NM_000450.2
**NFκB-p65**	GCGAGAGGAGCACAGATACC	AGGGGTTGTTGTTGGTCTGG	NM_001145138.1
**JAM-C**	GCAATCGAACCCCAGTGGTA	GTCACTGGCTTCACCTCAATTT	NM_001205329.1
**VE-Cadherin**	CACGCCTCTGTCATGTACCA	TGTACTTGGTCTGGGTGAAGA	NM_001795.5
**P120 Catenin**	GGGCCAAGAAGGGCAAAGA	GCTGAGGCTTCTAGGATGGC	NM_001085458.2
**ESAM**	TAAGCCCGCTGTCCAATACC	TGGGCCTTGCAGACATAGAC	NM_138961.3

Primer sequences utilized in mRNA analysis via qPCR. Primers were synthesized by Integrated DNA Technologies (Coralvile, Iowa, USA). All primers were first checked for suitably in target species using the NCBI NIH PrimerBLAST system (available at https://www.ncbi.nlm.nih.gov/tools/primer-blast/). Subject to their suitability the primers were then QC checked using the Sequence Manipulation Suite available at (www.bioinformatics.org/sms2/pcr_primer_stats.html) prior to their use.
